# Increasing the Local Relevance of Epidemiological Research: Situated Knowledge of Cattle Disease Among Basongora Pastoralists in Uganda

**DOI:** 10.3389/fvets.2018.00119

**Published:** 2018-06-07

**Authors:** Erika Chenais, Klara Fischer

**Affiliations:** ^1^Department of Disease Control and Epidemiology, National Veterinary Institute, Uppsala, Sweden; ^2^Department of Urban and Rural Development, Swedish University of Agricultural Sciences, Uppsala, Sweden

**Keywords:** participatory epidemiology, livestock, disease ranking, local knowledge, participatory research, participatory rural appraisal

## Abstract

Cattle disease can have severe negative impacts on the livelihoods of the poor, but still, animal disease management and outreach often remain suboptimal in low-income settings. In a study on Basongora pastoralists in Uganda, we examined local priorities, perceptions and practices regarding cattle disease, in order to improve outreach and disease control advisory work in such contexts. We also investigated how participatory epidemiology can be better equipped for gathering situated knowledge. Empirical material obtained in focus group discussions, interviews, participatory mapping, and wealth-ranking was used to perform a thematic, bottom-up analysis. The concepts of situated knowledge and embodied objectivity and insights from participatory research and interdisciplinary dialogue were applied to better embrace local perspectives. Cowdriosis, trypanosomosis, contagious bovine pleuropneumonia, East Coast fever and anthrax were high-priority diseases for participants. Lack of control over the animal health situation and money invested in treatments that did not guarantee recovery were of general importance for disease prioritization. Participants' descriptions of diseases sometimes diverged from textbook definitions. Co-infections, chronic and recurring infections and lack of access to formal knowledge were identified as important factors for differences between formal and situated knowledge. Paying attention to situated knowledge and particular context-specific issues such as proximity to a national park proved to be of special relevance for local understanding and experiences with disease. Another factor was the local importance ascribed to number of cattle, rather than production levels. These factors need to be taken into consideration when formulating disease control advice, as does the complex disease landscape. The results reveal the importance of moving research and advice beyond curing “knowledge-gaps” and creating different ways of understanding disease so that situated knowledge can be considered, and disease control improved.

## Introduction

**Figure 1:**
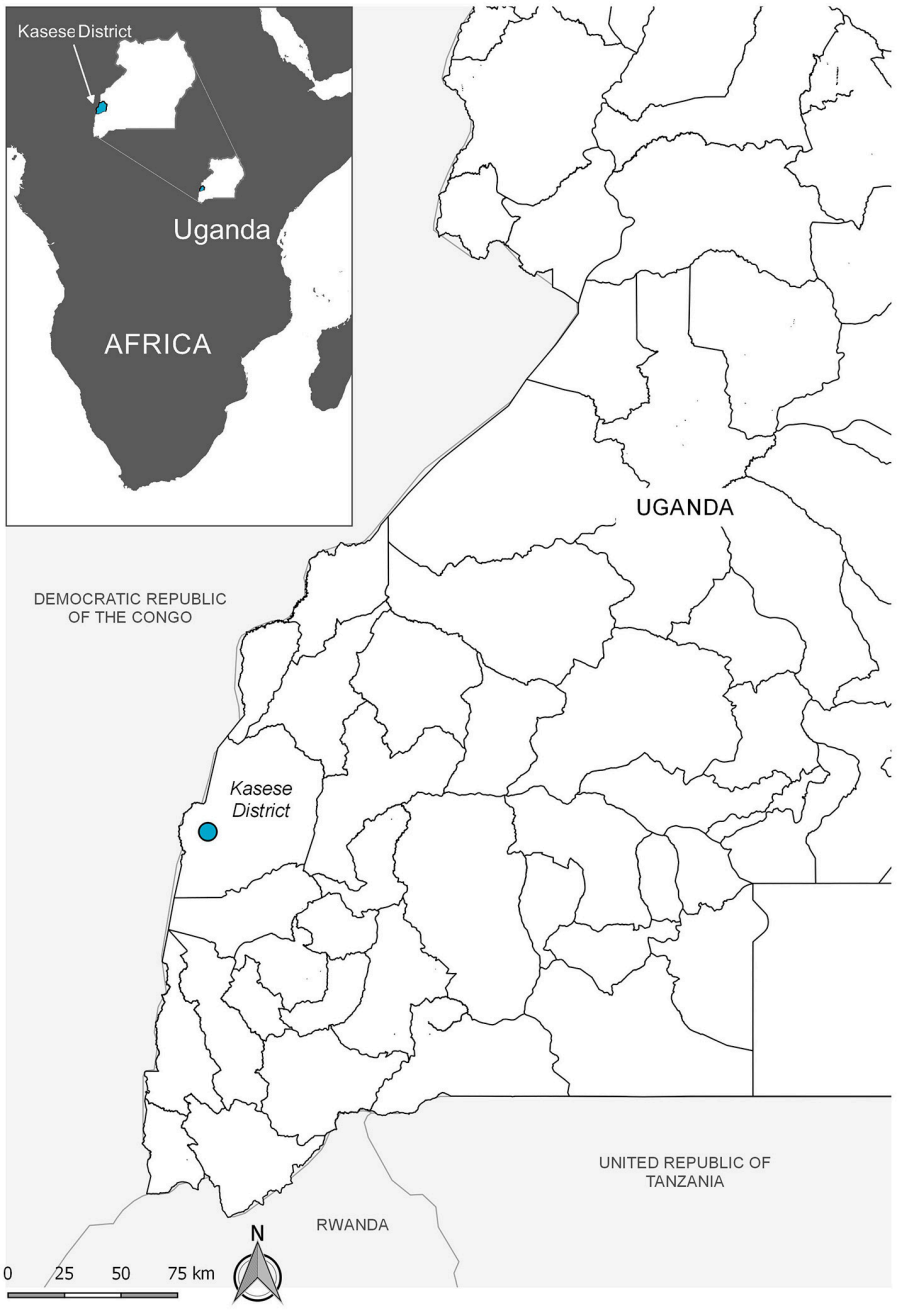
The study village, marked as a dot on the map, is located in the western part of the “cattle corridor” stretching through Uganda in a north-westerly direction.

Livestock are crucial for the livelihood security of many poor people. They provide valuable protein, manure, and draft power, but also function as social status symbols and walking banks (1). The embodied effects of animal disease thus often markedly increase livelihood vulnerability (2). This study was performed with Basongora pastoralists in Isaazi village, Nyakatonzi subcounty, Kasese district, south-western Uganda (see Figure 1).

Due to their dependence on cattle, pastoralist livelihoods might be particularly vulnerable to the impact of bovine disease. For the same reason, pastoralists can be expected to be more knowledgeable about these diseases and their treatment than other poor communities in similar contexts. In this study we sought to consider how the Basongora in Kasese district prioritize, understand and deal with cattle disease and the related constraints they face. Our aim was to start from local perspectives and priorities, and let these guide the study. Few such studies have been performed to date in veterinary medicine and epidemiology, Examples of the exception are an early participatory epidemiological (PE) study of animal diseases in pastoralist communities in Somaliland (3), and a later study on goat diseases in Turkana South District in Kenya (4). These studies both applied local perspectives to identify diseases of importance for the studied communities.

Based on the findings from the present study, we discuss how future research might better take into account local knowledge and priorities, and what the effects of doing so were in the present study. We also suggest some ways of making epidemiological research more fully embrace local perspectives and practices, and thus provide research findings of increased local relevance. Finally, we suggest some practical measures for improving outreach and adoption of disease management in these contexts.

### Researching situated knowledge of cattle disease

The importance of researchers and policy makers acknowledging that all knowledge is situated, and not simply regarding local ways of knowing and prioritizing as inferior, has been repeatedly emphasized [e.g., (5, 6)]. However, recent research shows how acknowledging other forms of knowing than formal “textbook” knowledge has arrived later in veterinary medicine than in many other academic disciplines, and that such acknowledgement can have significant positive effects on the dialogue between veterinarians and farmers (7). For example, applying very detailed knowledge about a disease and associated recommendations about its treatment produced by veterinary researchers without local engagement, might not be wrong *per se*. However, such approaches will likely produce knowledge that is not well anchored in the local situation and does not accurately acknowledge complex disease ecologies and economic constraints limiting treatment possibilities. Such decontextualized knowledge will be difficult for local people to act on (7).

We argue here, that if veterinary research and practice take the approach to knowledge not as an object to be gained or not, but as something situated and locally specific, it will be better equipped to understand local accounts of disease, including how and why they might differ from textbook descriptions. Such acknowledgement would facilitate both research and practical veterinary work [see (7) for a similar reasoning]. One of the early writers on this is Haraway (8), who describes how modern science has colonized “objectivity” as detached from context, and universally applicable. By slicing up reality and dividing responsibility for understanding the world into different disciplines, very detailed and seemingly “objective,” but highly decontextualized and selective, accounts of the world are created. Haraway (8) points out that these accounts of the world, like any other knowledge, are partial and situated but claim to be general, thereby particularly excluding knowledge and realities of marginalized groups in society. The term “embodied objectivity” reflects the fact that objectivity is never detached and neutral, but must be judged in its context [see also e.g., (5, 6)].

One strategy for facilitating this openness to different ways of knowing has been to engage in interdisciplinary dialogue (9, 10) and to employ participatory methods (6, 11). Participatory methods aim at making policy and research more sensitive to local conditions (12). By doing so it can have a significant impact in attuning development work and research to poor people's realities (13). In this way participatory methods can also facilitate that implementation of research findings and policy interventions are grounded in priorities and needs of the local people. PE in veterinary science has been developed as a tool for collecting epidemiological data in contexts where conventional quantitative data are unavailable. However, recent research has shown that the focus on being accepted by the conventional veterinary research community has led to “participation” in PE, and the resulting relevance of the findings to local people, being rather limited (14). In this study, we as authors combined our expertise in veterinary medicine and rural development studies, respectively, while remaining equally open to local competence, drawing on participatory methods.

## Materials and methods

Data collection was designed to embrace local perspectives on cattle disease. We performed focus group discussions (FGDs), individual interviews, participatory wealth-ranking and mapping. Participants were selected on the basis of purposive sampling strategies (15). All interviews were guided by a pre-defined topic guide outlining broad topic areas, while remaining open to inclusion of additional topics by participants. Before implementation of the study the local research team, consisting of facilitator, note-taker (both veterinarians) and an interpreter, jointly translated the interview topic guide from English to Lutoro/Rusongora. A pilot FGD was conducted to test the set-up and the local relevance of the questions. The participants in the pilot FGD were from a village neighboring the study village and recruited in the same way, with the same requirements and procedures, as for FGDs included in the study. Results from the pilot FGD were not included in the results. The topic guide can be found as Supplementary Material 1.

### Data collection

Following the pilot, eight FGDs, four with women and four with men, were performed. These groups were convened by a key informant working for a local non-governmental organization in the study village and residing in an adjacent village. The requirements for participation in FGDs were that participants lived in the study village, were over 18 years and owned or tended cattle. Groups of at most nine participants were organized and each new group consisted of people who had not previously participated.

In the beginning of each interview and FGD the research team informed about the study and its objectives, especially pointing out that it was a research project and not a need assessment or similar, with possible immediate benefits for the community. All respondents were asked for their oral or written consent (including for audio recordings and photos) and informed that they could refuse to answer questions and withdraw from the group at any time. The facilitator followed the topic guide while being sensitive to participants' wishes to express concerns and comments outside this frame, and ensured that the discussion was not dominated by one or a few individuals. The authors intervened and gave feed-back if deemed necessary. All participants spoke Rusongora, while the interpreter and the facilitator spoke Lutoro. Lutoro and Rusongora are sufficiently similar for translation to work smoothly, but inevitably some detail may have been lost. Detailed notes were taken throughout the field work. The discussion was simultaneously translated to English and the translation recorded on audio-tape for back-up, but not transcribed verbatim. Notes taken by the note-taker and both authors, as well as the audio-tape recordings, were frequently compared and discussed with the research team and key informants. Quotes used in this paper should not be seen as exact translations, but as illustrations intended to give life to the findings.

**Figure 2:**
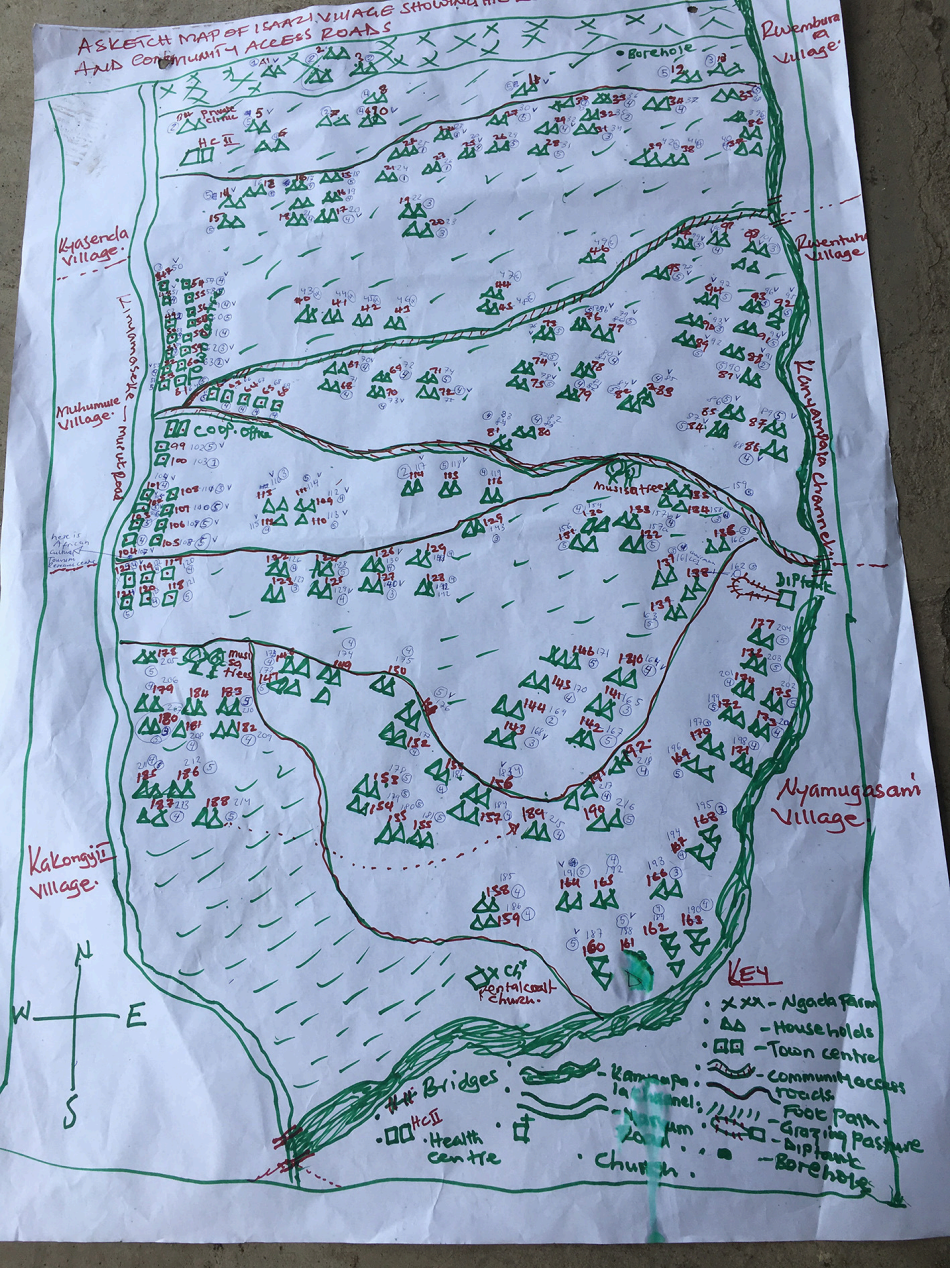
Map of the study village indicating all households and their wealth rank. Households are numbered 1–199 in plain numerals. Wealth rank is indicated as 1–5 in encircled numbers. Households marked √ participated in the first eight FGDs.

Participatory mapping (16) of the village depicting all households was conducted (see Figure 2). The group performing the participatory map and wealth ranking consisted of five men identified by the key informant for their knowledge of the village and of all households. They had not participated in the FGDs. The participatory mapping improved the research team's understanding of the local geography, helped identify geographical areas of the village covered in initial focus groups and was used as a basis for subsequent wealth ranking. The wealth ranking, designed with inspiration from Jacobson (16), aimed at capturing local perspectives on poverty and wealth and identifying the relative wealth of each household using the participatory map. There was significant agreement in the group about factors deciding wealth rank. These were:

- Education of children.
- Private ownership of land.
- Number of cattle.
- Household monetary income.
- Quality of dwelling house.

Based on these factors, participants agreed on five different wealth ranks, which were subsequently cross-checked in a FGD with five women. This revealed substantial correlation between the factors prioritized and the overall importance of cattle, but the women added the importance of wife and children being well fed.

The mapping indicated that 70 out of a total of 199 households participated in the initial eight FGDs. There was no notable difference in the distribution of wealth ranks of the households participating and not participating in the initial eight FGD groups. However, one geographical area where many widowed or divorced women live emerged as under-represented in the initial eight focus groups. We therefore conducted one more FGD with five women from this area of the village. The participants for this FGD were recruited with the same instruction as for the initial eight FGDs, but restricted to women living in the indicated area. This FGD also served the purpose of cross-checking the factors deciding the wealth rank, as described above. We also conducted one additional FGD with seven (male) cattle care herders owning few or no own cattle, and four additional interviews with seven young men herding other people's cattle, as we suspected that the perspectives of this group might not have emerged in the FGDs. The herders were encountered and approached while the researchers walked around the study village and recruited for an immediate FGD or interview. This approach was selected as it was difficult to make herders leave their duties and take part in a scheduled FGD. However, the FGD and interviews with herders did not indicate that these participants had different knowledge and experience of cattle disease than cattle owners.

With the overarching research question as a guide, we conducted a thematic, bottom-up analysis where we let the empirical data guide the categories emerging. In both data collection and analysis, we aimed at preserving the diversity of perspectives emerging, rather than forcing consensus. Literature on the local social and ecological conditions affecting Basongora livestock keepers, and of the diseases and vectors mentioned by interviewees, facilitated analysis of the local empirical material and provided grounds for dialogue between local and non-local knowledge on cattle production and disease.

## Results

Cattle disease was a topic that prompted significant engagement in the interviews, indicating the significant of cattle in the Basongora culture. Number of cattle owned was central for perceived status and wealth in the community, and even households ranked among the poorest still had a few cattle, indicating the priority given to investing in cattle. However, similarly to many other traditional cattle-based communities (17), it was the number of cattle that was important and production levels were not prioritized. Cattle were frequently kept to old age (up to 16 years) and cows were described to produce up to 15 calves in their lifetime. Cows were further described to calve the first time at 6 years of age and producing approximately 2–3 L of milk per day during the lactation period.

### Situated knowledge of cattle disease in the study village

During the FGDs, participants were asked to list all diseases they had observed in their cattle in the past 2 years. The limit of 2 years was set to give a period sufficiently near in time for participants to remember and sufficiently long to capture a wider range of diseases of relevance. However, we did not relate this time span to any other specific events in the community that could have helped the participants define it more exactly, and thus it should be taken as a rough indicator. A total of 38 different cattle diseases, syndromes, signs or external parasites were mentioned (Table 1). The fact that many of these are not diseases in a formal sense indicates the broader perception of disease in the community, the general lack of a strong link between formal and informal veterinary knowledge and lack of access to veterinary services. Since 1999, every sub-county in Uganda is expected to have a government-employed veterinarian. Kasese district is divided into 23 rural sub-counties and six town councils/divisions (18, 19). While Nyakatonzi is one of the sub-counties that have employed a veterinarian, this veterinarian does not live in Isaazi village. However, a private veterinarian is residing in Isaazi. Local estimates and our own calculations based on wealth ranking indicate that Isaazi villagers own 8,000–10,000 cattle, excluding calves. Since Isaazi is one of 11 villages in Nyakatonzi sub-county, the total number of cattle clearly exceeds the amount that one or two veterinarians could handle. Thus, participants to a large extent have to manage disease and other production challenges without consulting animal health professionals. Of the 38 diseases mentioned, 12 were described as being the most important in one or more FGDs and five (cowdriosis, trypanosomosis, contagious bovine pleuropneumonia (CBPP), East Coast fever (ECF) and anthrax) were mentioned particularly frequently (Table 2). There was no obvious difference in interview responses between women or men, or between cattle owners and herders, regarding the diseases mentioned or their prioritization. Below we provide brief textbook-type descriptions of these five diseases, followed by their Rusongora names, local descriptions, including their signs and causes, and participants' stated reasons for rating a particular disease as most important.

**Table 1:** Exhaustive list of all cattle diseases, syndromes, signs or external parasites mentioned in focus group discussions (FGDs).

Disease^*^	Number of FGDs mentioning the disease^*^
Anthrax, cowdriosis, East Coast fever (ECF), trypanosomosis	10 (= all)
Contagious bovine pleuropneumonia (CBPP)	9
Foot and mouth disease, worms	8
Fever, lumpy skin disease	7
Ephemeral fever	6
Anaplasmosis, tuberculosis	5
Diarrhoea, tetanus	4
Eye-worms	3
Cough, brucellosis, pink eye, tick fever	2
Abscesses, bloody diarrhoea, constant urinating, constipation, fever and cow goes blind, head shaking, high fever and dry faces during dry spell, laminitis, mastitis, papillomatosis, photosensitivity, rhinderpest, ring womb, ring worm, small elephant flies, standing hair coat, still births, ticks, unknown disease: rotten intestines at slaughter	1

* *Disease (including diseases, syndromes, signs or external parasites) names are those given by the participants, directly translated into English*.

**Table 2:** Diseases, syndromes, signs or external parasites ranked among the top five most important in at least one focus group discussion (FGD).

Disease^*^	Number of FGDs where the disease^*^ was ranked top five
Cowdriosis, trypanosomosis	9
Contagious bovine pleuropneumonia (CBPP)	8
East Coast Fever (ECF)	6
Anthrax	5
Diarrhoea, fever, tick fever, worms	2
Cough, bloody diarrhoea, eye-worms	1

* *Disease (including diseases, syndromes, signs or external parasites) names are those given by the participants, directly translated into English*.

#### Cowdriosis

Cowdriosis, or heartwater, is caused by the bacteria *Ehrlichia ruminantium* and is spread by Bont ticks (*Amblyomma* spp.) (20). The disease is endemic in many parts of Africa and causes fever, nervous signs, diarrhoa and ultimately death (21). The Rusongora name for cowdriosis is *omutwe*, literally meaning headache. All FGD participants described the signs of cowdriosis as cattle getting a stiff neck or head leaning to one side and the animal starting to move in circles, isolating itself and turning mad. Many other, less specific, signs of the disease were also mentioned, and it was pointed out repeatedly that it was difficult to diagnose the disease in time to enable successful treatment.

Although all FGDs identified cowdriosis as a tick-borne disease, tsetse flies (*Glossinia* spp.) and small elephant flies (*Tabanidae* spp.) were also mentioned as disease vectors. Other factors mentioned as causing the disease were prolonged drought and lack of feed, animals being struck too hard by the herder or animals fighting with other animals. Lack of access to veterinary services, not applying acaricides according to recommendations and treatment failure of acaricides were also mentioned as reasons for the disease. Cowdriosis was regarded as important because it is common and causes (sudden) death. Both preventive and curative measures were mentioned as being ineffective and expensive. The clinical signs from the central nervous system were mentioned as both dangerous to manage and economically damaging, as infected cattle stray and get lost, preventing sale or consumption of the meat. Infected wild animals and vectors from the nearby national park were mentioned as complicating control of the disease.

#### Trypanosomosis

In East Africa, trypanosomosis in cattle is generally caused by *Trypanosoma brucei brucei, T. congolense* or *T. vivax*, and is transmitted by tsetse flies (*Glossinia* spp.). Infection can cause various signs such as lymphadenopathy, anemia, anorexia and death (22). Signs can present along a range from acute to chronic (21). The Rusongora name for trypanosomosis is *ekipumpuru*, which translates directly as emaciation.

The signs of trypanosomosis mentioned in the FGDs were many and varied. Many related to production losses, such as milk drop, giving birth to weak calves and abortion, but also e.g., diarrhoa, standing hair coat, lack of appetite, weight loss, eye problems and swelling around the neck. Some participants also mentioned that the signs are vague and that the disease weakens the immune system, which might mean that simultaneous infections make it particularly difficult to diagnose the disease.

Seven out of 10 FGDs mentioned tsetse flies as causing trypanosomosis, but six FGD also mentioned ticks as insect vectors. Many participants further emphasized the role of elephants (from the national park) in disease transmission. Elephants kicking the soil and making holes where water collected and cattle drinking together with elephants were mentioned as causing disease transmission. Hunger, limited pasture, and prolonged drought were also mentioned as causing the disease. Some FGD participants associated increased incidence during drought with a disease-causing agent in the soil.

Trypanosomosis was regarded as important because it causes death, particularly in calves. It was also mentioned that this disease often recurs after treatment. Economic impact related to impaired production, failed reproduction, difficulties in selling infected animals and costly treatment were also frequently mentioned. The difficulties in selling infected cattle were specifically related to the impaired body condition, probably further reflected in the Rusongora name for the disease. Ticks, tsetse flites and vertebrate vectors from the national park were mentioned as complicating factors in controlling the disease.

#### Contagious bovine pleuropneumonia (CBPP)

Contagious bovine pleuropneumonia is covered by animal health laws in Uganda and its control is under governmental responsibility (23). It is caused by *Mycoplasma mycoides* subsp. *mycoides* and causes a very serious and contagious respiratory disease (21). The Rusongora name for CBPP is *kihaha*, which is closely related to the word for lungs (*ekihaha*). CBPP was particularly described as causing cough. Other signs mentioned were discharge from nose and eyes, lethargy, loss of weight, diarrhoa, standing hair coat, fever, abortions, and milk drop. Several post-mortem signs were also mentioned (particularly by the men, as women do not participate in slaughter) including damaged lungs and the lungs being attached to ribs and to other organs.

Many participants mentioned that CBPP can spread through infected cows entering a herd or passing nearby. Transmission occurring through wildlife (from the national park), and through humans who had stepped in infected cow dung was also mentioned. However, the particular pathogen was not known by the participants.

CBPP was regarded important because it is common, causes many deaths, is easily transmitted and requires treatment that is not available locally. Economic impacts arise from loss of value of cattle, and from trade restrictions due to quarantine (“*quarantine causes loss of income because you cannot sell milk or sell cattle that is needed to pay for school fees*”). The post-mortem signs also reduce the value of the meat and cause fear of zoonotic disease transmission, probably as a result of the damage the disease causes to internal organs (although it can be noted that in fact the disease is not zoonotic).

#### East coast fever (ECF)

East Coast fever is caused by the parasite *Theileria parvum* spread by the brown ear tick (*Ripicephalus appendiculatus*) (20). It is endemic in east Africa, often causing fever, enlarged lymph nodes, dyspnoea, wasting, and diarrhoa, followed by death (21). The Rusongora name for ECF is *omuswija*, which translates as high fever.

Like trypanosomosis, ECF was described with a variety of symptoms. Many participants said that it was more common in calves, or even that it only occurred in calves. Signs of disease mentioned included fever, swollen lymph nodes, nasal discharge, and swollen eyes, standing hair coat, cough, labored respiration, diarrhoa, inability to stand up and anorexia.

Only five out of 10 FGDs said that ECF was caused by ticks. Some also mentioned flies in general, and tsetse flies in particular, as disease vectors. Dry spells and too much wind and sunshine were also mentioned as causing the disease. Calves drinking too much milk or irregular milking (i.e., irregular milk availability for calves) were repeatedly mentioned as factors causing the disease. Some also said that the disease could spread by milking infected cows. ECF was regarded as important by participants because it is common and causes death, especially in calves (“*Calves are cows of tomorrow, if calves die that is bad*”). Sudden appearance, difficulties in diagnosis and the need for treatment to avoid a fatal outcome were also mentioned, as was fear of zoonotic infection potential via milk (“*If you take the milk from a cow with fever also the humans get sick, but you cannot stop taking milk, it is the delicacy*”).

#### Anthrax

Anthrax is a fatal disease in cattle, often presenting with per-acute death. It is caused by spore-producing bacteria *Bacillus anthracis*, the spores of which can survive in soil for a very long time. The spores are often unearthed in extreme weather conditions such as droughts or floods, or a combination of these (21). The Rusongora name for anthrax is *kakooto*. The etymological background to this name could not be clarified, but it is possibly related to the word for “enlarged.”

Anthrax was particularly described by its sudden appearance. Many said that there were no signs before death, while others mentioned swelling of the body and bleeding from nose and anus before or just after death. In particular, men also reported several post-mortem signs, including the meat looking “*as if it were boiled*,” enlarged spleen, no rigor mortis, watery blood and blood coming out of body orifices.

Many participants mentioned that anthrax comes from the soil, especially during droughts. Those who did not identify the soil as the disease source were still aware that anthrax appears especially during droughts, and that it comes somewhere from the pasture. It was well known that meat from infected cattle is a danger to public health. Anthrax was regarded as important because of its deadly outcome, the sudden onset (“*Anthrax can attack without noticing, you only realize as blood is oozing out of mouth and anus*”) and by affecting seemingly healthy cattle. The zoonotic potential and the economic impact from not being able to consume or sell the meat were also mentioned.

### Local priorities of cattle disease

As seen in the sections above, many of the reasons given for the relative importance of particular diseases were general, and more related to the participants' situation and the context than to the specific diseases. Such more general aspects are described below.

The aspects of the diseases that influenced perceptions of their relative importance can be grouped into themes relating to: epidemiological parameters and expected final outcome of the disease, prospects for success of available treatment, economic impact, clinical signs, causes of the diseases, the national park, acaricides, and uncertain elements (i.e., if the cause, diagnosis or treatment was unknown to the participants). In more detail, and using epidemiological terms, the first theme relating to the relative importance of a disease could be described as disease incidence, prevalence, contagiousness, hereditary potential, and case fatality rate. Aspects of the outcome of disease, notably death or a chronic/progressive disease course, added to the relative importance of a disease. A disease appearing suddenly or death occurring without previous signs were factors contributing to the relative importance. Such aspects make diseases difficult to prevent or control and add to livelihood vulnerability. For similar reasons, the expected success of treatment, if a disease recurs after treatment, if the treatment does not cure the disease and if the animal dies even when treated were repeatedly emphasized. In this regard the availability of drugs and the problem with inefficient acaricide treatments were frequently raised. Economic impact (e.g., cost of treatment, possibility to sell the meat, market value of cattle, zoonotic potential hindering consumption and trade, and diseases imposing quarantine measures preventing trade of cattle and their products) were also mentioned as important. However, economic impact went well beyond monetary value at point of sale and could be described in terms of: (1) Maintaining the herd (rather than e.g., being able to sell animals) and (2) spending money on ineffective treatment. Again, underlining the value of herd size, disease in calves was often mentioned as particularly devastating owing to their value as future replacements for cows. With regard to ineffective treatment, in particular the problem with ineffective or “fake” acaricides on the market was repeatedly stressed, also when talking about diseases that are not tick-borne (“*acaricides are ineffective even if we spray twice weekly*,” “*we lost a lot of money because the acaricides did not kill the ticks*”).

### Local disease management

Participants reported that they often did not consult animal health professionals but treated their cattle themselves or sought help from other community members. This was especially mentioned as being the practice for the more common diseases such as cowdriosis, trypanosomosis, ECF, parasites, fever, and diarrhoa. In these cases, therapeutic drugs were obtained from the local market, drug shop or agriculture input provider. Furthermore, the long tradition, culture and experience of cattle keeping in this community were mentioned as providing a base of knowledge about treating cattle diseases. One of the key informants explained that everyone in the village had significant knowledge about cattle production, different diseases, and their treatment, “*but what differs between people is the possibilities to do something about the disease*.”

Some traditional treatments options were mentioned, such as using particular plant extracts for treating cowdriosis and trypanosomosis. At the same time, local treatments seemed overall to have significantly declined with the introduction of formal medicines, even when these did not function satisfactorily. For example, despite the very frequent complaints about ineffective acaricides, other means of controlling ticks, such as hand-picking or smearing plant extracts, urine or waste oil, were mentioned as not being in use anymore.

Many participants mentioned that they would contact the local village veterinarian, or the government veterinarian, if their own treatment did not succeed, for diseases they did not recognize or for some specific diseases (CBPP, foot, and mouth disease, anthrax, lumpy skin disease). However, despite Nyakatonzi being one of the sub-counties in Kasese district that have a government veterinarian employed, and despite a private veterinarian residing in the village, many villagers reported difficulties in accessing veterinary healthcare. Apart from lack of access, avoiding costs for paying the veterinarian was mentioned as a reason for treating cattle themselves or asking other community members for help before consulting animal health professionals. As the veterinarian was often called out only after local attempts at treatment had failed, the success rate of veterinary treatments is also likely to be low.

## Discussion

The results and available literature indicate several reasons for diversions between textbook and local disease descriptions. These include co-infections, chronic, intermittent and recurring infections and lack of local knowledge on disease-causing agents, leading to local understanding, and classification of diseases focusing on clinical signs. As an example, the clinical signs used to describe trypanosomosis were especially varied. In a study by Catley et al. (24), agro-pastoralists in Sudan described trypanosomosis as a chronic wasting disease, leading those authors to conclude that the varied signs described might reflect co-infections with several endemic diseases, as might also be the case in our study. Another explanation for the varied, and somewhat vague, descriptions of clinical signs might be that the cattle suffer from more or less constant under-nourishment (25), leading to a general weakened immune response, making differential diagnosis difficult. Although not discussed as such by the participants, we for example interpret the local accounts regarding cattle reproduction and milk production as harsh ecological conditions and disease pressure causing significant constrains on productivity.

In a study of local knowledge on tick-borne diseases (TBDs) in cattle among Karamoja pastoralists in Uganda, participants described co-infections with several TBDs such as anaplasmosis and ECF, leading to the conclusion that such co-infections might lead to under-estimation of the true prevalence of disease (26). Co-infections also affect disease management and the local relevance of disease control advice. Participants in our study frequently discussed treatment failure of acaricides as a reason for failed prophylaxis of trypanosomosis, despite the insect vector being tsetse flies, not ticks (22). Both single and combination preparations (effective against ticks and tsetse flies) were used in the study village, but this difference in vectoricid-range was never mentioned by participants. Rather than interpreting this as local misrecognition of the disease-causing agent, we concluded that if cattle are concurrently exposed to tsetse flies carrying *Trypanosoma* spp. and several species of ticks (20) carrying one or several TBDs, a sub-clinical infection by any TBD might exhaust the animal's immune system, making it succumb to clinical trypanosomosis. The local emphasis on acaricides for treating non-TBDs might also reflect a general wish to have access to better disease treatments. In the same way, the local connection made between trypanosomosis, reproductive failure and abortions might be caused by local failures to distinguish between trypanosomosis and diseases more commonly associated with signs from reproduction system such as brucellosis, or by co-infections making the signs less obvious. Brucellosis was only mentioned in two out of ten FGDs, and never mentioned as an important disease. In recent findings from the same area by Wolff et al. (27) the prevalence of brucellosis was high (40%).

Another example of how a broad exploration of situated knowledge of animal disease could contribute to more robust research findings on local disease-related challenges and assist in potential identification of new diseases was the case of “fever.” Participants described three different syndromes of “fever”: fever, tick-borne fever and ECF. While ECF was acknowledged locally as an important disease, the descriptions often did not comply with the textbook description. It was described as tick-borne in only half of all mentions and was frequently discussed as particularly affecting, and being more serious in, calves. However, under endemic conditions, young animals are at least partly protected by maternal antibodies and calves are thus often described as being less prone to clinical disease (28). The accounts in our study included high neonatal death rate, diarrhoa and overall high case fatality rate in calves. This led us to conclude that the disease described as ECF in calves might actually be another disease. Methodologically, it must be noted that we failed to be sufficiently thorough when discussing with the local research team about how to translate the diseases and to allow for an openness about that there might not be a one to one relationship between local and formal, as well as Rusongora/Lutoro/English disease names and meanings.

During FGDs we noted a tendency for the facilitator to force participants' descriptions of syndromes into scientifically accepted disease nomenclature that could have caused misclassification. More thorough preparation and discussions with facilitator and translator about local names and meanings of different diseases could have avoided this. As ECF in Rusongora is translated as high fever, it is likely that this term groups together several reasons for high fever, and thus that some of what was reported to us as ECF might in fact have been a different form of high fever in cattle. Two FGDs prioritized “diarrhoa in calves” as one of the five most important diseases. In these two groups, the syndrome was discussed at length and seemed to have had very negative impacts for the participants. The syndrome translated as “ECF in calves” might equally have been translated as “high fever in calves” and might actually be the same disease as “diarrhoa in calves.” These two accounts of a disease new to the study community that presents with high case fatality rate in young calves can be triangulated with recent findings reporting high prevalence of bovine viral diarrhoa virus in the same area (27). It can be noted here that if our study had only been about one disease, e.g., ECF, our conclusion regarding the local reports on ECF might instead be only that there is a lack of knowledge on ECF in the study village. Acknowledging the possibility of several diseases and symptoms being incorporated within the formal name of one single disease, rather than just interpreting local inconsistencies in the characterization of ECF requires openness to different ways of understanding and classifying disease and to the possibilities of reasons other than lack of knowledge behind these local accounts.

### Lessons for disease treatment and advice

As noted in almost all local disease descriptions, wildlife from the nearby national park was perceived as a significant local problem for disease management; one that the pastoralists also felt that they had limited influence over. The Basongora in Kasese are together with the Karamojong to the north among few pastoral groups who still practice communal grazing in Uganda. However, their grazing land has shrunk over time due to continued competition for land from neighboring farming communities, national parks, and commercial cotton production (29). With the establishment of Queen Elizabeth National Park in 1954, which Isaazi borders, villagers lost a significant part of their grazing lands. The park still causes ongoing conflicts between pastoralists and wildlife, as mentioned frequently in interviews and confirmed by other studies in the area (30, 31). The frequency of the complaints about the national park in our interviews are clearly in part strongly influenced by the ongoing land-use tensions, which was also reflected in the wish for private ownership of land, described as a solution for secure grazing and limiting disease transmission between herds. While private ownership of land of enough size to secure own grazing will probably never be achieved, this wish also reflects a desire to have more control over the entire animal health situation. Indeed, in discussions on key challenges in disease prevention and treatment, the experienced lack of control over disease cause, prevention and treatment was evident in several ways, as was the frustration that money invested in treatment did not guarantee recovery. Given the significant livelihood vulnerability caused by animal disease and the perceived lack of control over the disease situation, local suggestions for improving animal health focused to a large extent on structural investments by the government to reduce local vulnerability (e.g., building a dip tank, vaccination, fencing the national park, providing veterinary services, and drugs). These suggestions are largely in agreement with those by Coffin et al. (31) and Byaruhanga et al. (26) for other parts of Uganda. Our study also showed common community willingness to participate and sustain infrastructures for disease control. Local residents had for example formed a producer group to restore the local dip tank. The local emphasis on the need for structural support, while at the same time seemingly not complying with some of the existing veterinary advice, can be interpreted such as that this advice were not easily adopted under local circumstances, and that structural support was judged locally to have the potential for more significant impact [see also (16, 32)]. In the present study there were examples where local tradition and livelihood constraints clearly made it difficult to act in ways that would reduce disease transmission, even if the knowledge about how to do it was there. The problems with disease transmissions from the national park is one such example. Also, there was widespread recognition among participants in this study of milk as a disease-transmitting agent but, despite this, there was evidence that recommended withdrawal times for milk during disease and treatments were not followed, because milk is an important income and an appreciated delicacy.

Some of the local lack of control over the disease situation could also clearly be reduced with more access to information of disease prevention and treatment. Like pastoralists in other parts of Africa (4), participants in this study in most cases treated sick cattle themselves, without consulting a veterinarian. The Basongora have a long tradition of keeping cattle and associated knowledge of signs of diseases. At the same time, as indicated above, local knowledge of disease-causing agents and associated evidence-based treatment is often limited, and co-infections and generally low health status of animals further complicate diagnosis and treatment. There was a strong desire amongst many participants to learn more about on how to control and treat diseases. One example of this was the frustration with the local lack of solution, and wish for us to have an answer, to the problem with diarrhoa in calves. The local importance of cattle for providing protection against vulnerability and calves as the future economic security clearly made this a pressing problem. In general, participants repeatedly emphasized the importance of us reporting back our findings to them and asked us many questions about correct ways of treating diseases. As discussed in the final section, the results high-light the importance of such information being given in ways that makes local sense and is possible to act on.

### Methodological lessons for making epidemiological research and practice more attuned to local people's realities

Despite the complex disease landscapes in many low-income countries, few studies discuss co-infections and related implications for animal health, disease control and poverty reduction. Furthermore, even with increased acknowledgement within epidemiological research of local disease prioritizations, especially through the growing field of PE (33), studies frequently remain focused on single diseases. Demands by funding bodies and the wider research culture for concrete and timely deliverables are important reasons why many studies have a pre-set and often rather narrow focus. In some cases, participatory methodologies are used as a first “scoping” stage, to set more detailed priorities for further research or development activities (4, 34, 35). Other studies focus on singling out “the most important disease in the studied community,” signifying a compartmentalistic view of animal management and health grounded in an illusion of a healthy animal being struck by a solitary disease event (36). The reality, according to our findings and those of others (37), is rather the opposite: co-infections, sub-clinical disease and diseases recurring due to therapy failure. Consequently, factors contributing to the relative importance of specific diseases were in many cases not disease-specific, but general. Moreover, when participants seemingly talked about a specific disease, it was apparent that they were frequently describing situations with co-infections. Accepting this complex disease landscape has implications not only for the questions we ask while doing research, but also for the answers we give in the form of outreach and advisory services. Consequently, finding all the scientifically relevant answers regarding single diseases might not have the highest priority for communities. Instead, more general actions that can address the over-arching health status of the herd and thus prevent sub-clinically infected individuals from succumbing to clinical disease might be more relevant. This might include improving biosecurity and feeding as well as other preventive measures such as immunization and acaricide or ecto/endo-parasite treatments.

In addition, as revealed by our investigation into the meaning of local disease names, local classifications of diseases are likely to differ somewhat from textbook definitions, as exemplified by ECF locally meaning “high fever,” which is likely to include ECF and other diseases causing high fever. Conventionally, triangulation is the recommended method for cross-checking local accounts of disease (33). This involves both cross-checking local descriptions of disease syndromes with key informants, and biological sampling to arrive at scientific disease names. While such approach is important for ensuring that one actually collects medical data and oral statements on the disease intended, it does not necessarily allow for an openness to local knowledge and classifications of diseases that do not fit neatly with formal scientific classifications. In this study we could have been more thorough in the preparatory work with the local research team to allow for such openness. The facilitator and note-taker in the present study were both veterinarians. This facilitated the translation of animal health terms and disease names from Lutoro/Rusongora to English, and was a valuable contribution to the triangulation process involving participants' account and official disease reporting. However, it could also have introduced a “professional filter” to what part of the discussion was conveyed and what diseases were noted down. Our results in this regard highlight the importance of continuous intense dialogue with facilitators and interpreters, preventing coercion of local accounts of disease into known nomenclature, and to the value of an open research focus in order to fully comprehend situated knowledge.

The obvious local need and wish for more information on how to deal with animal disease must be addressed. Like Coffin et al. (31), we emphasize the importance of studies claiming to be participatory taking the time to report results back to participating villagers in locally relevant ways. Several participants in this study expressed frustration about having been part of past research projects on cattle diseases and never being told the results. The present study is part of a larger research project studying tick-borne diseases on cattle in Uganda (Swedish Research Council Dnr 2016-05705). As part of this project we will report back the joint findings from the project during 2019. Our study can be used to emphasize the importance of information being locally appropriate, given in forms that makes sense in local terminologies, and that can result in concrete actions possible to implement.

## Statement of animal rights and research ethics

Data was collected from interviews, observation and participatory actives in the study village. All respondents were asked for their oral or written consent and informed that they could refuse to answer questions and withdraw from the interview at any time. All individuals are anonymized.

Ethical clearance for the study was approved by Uganda National Council for Science and Technology (ref A580) and Makerere University School of Veterinary medicine and Animal Resources Research Ethics Committee (SVARREC 03/2017).

## Author contributions

EC and KF contributed equally to all parts of the study (study design, construction of the interview topic guides, fieldwork, data analysis, drafting and writing the manuscript).

### Conflict of interest statement

The authors declare that the research was conducted in the absence of any commercial or financial relationships that could be construed as a potential conflict of interest.
