# Supplementary material for: Increasing the Local Relevance of Epidemiological Research: Situated Knowledge of Cattle Disease Among Basongora Pastoralists in Uganda
Source: Front Vet Sci. 2018 Jun 7;5:119. doi: 10.3389/fvets.2018.00119 (PMC6008553; doi:10.3389/fvets.2018.00119)
Supplement: Supplementary file 1 [file Data_Sheet_1.DOCX]

## Focus group and individual interviews

1. Present the project and its objectives and the project team (Erika)
2. Explain the consent form and the confidentiality (Masinde)
3. Fill in back ground data sheet (Samson)
4. Start the discussion (Masinde)

## Material needed

Back ground data sheet, flip chart, scotch tape, markers, tick cards, note book, pen, dictaphone, sodas and water, bisquits

## Interview/topic guide

### Warm up

Do you have/own/take care of cattle?

### Disease perception

Do you have problems with disease in the cattle?

Which disease have you encountered in the cattle in the last two years? (list all mentioned disease on flip chart)

Of these, which five diseases do the group consider most important, and why?

Ranking exercise of five disease that the participant judges as most important (rank 1-5, can use list with all diseases)

Describe signs and what is causing the 5 selected diseases

### Disease management

What do you do when your cattle are sick? (Poke for details, do not only accept “I call the vet”)

### Ticks

Are ticks a problem for the cattle?

Are ticks causing any of the mentioned diseases? (all, not only the top 5)

Can you describe different ticks?

Are different ticks related to different diseases? (pair tick from own description or tick card with diseases on list)

### Tick management

How do you manage ticks today?

How would you like to manage ticks in the future?

### Rounding up

Anything else you would like to discuss?
